# Advances and challenges in targeting IRF5, a key regulator of inflammation

**DOI:** 10.1111/febs.14654

**Published:** 2018-09-21

**Authors:** Hannah Almuttaqi, Irina A. Udalova

**Affiliations:** ^1^ Kennedy Institute of Rheumatology University of Oxford UK

**Keywords:** autoimmune diseases, inflammation, interferon regulatory factor 5, therapy, transcription factor

## Abstract

Interferon regulatory factor 5 (IRF5) belongs to a family of transcription factors, originally implicated in antiviral responses and interferon production. However, studies conducted in different laboratories over the last decade have placed IRF5 as a central regulator of the inflammatory response. It has become clear that IRF5 contributes to the pathogenesis of many inflammatory and autoimmune diseases, such as rheumatoid arthritis, inflammatory bowel disease and systemic lupus erythematosus. Given the role of IRF5 in physiology and disease, IRF5 represents a potential therapeutic target. However, despite a significant interest from the pharmaceutical industry, inhibitors that interfere with the IRF5 pathway remain elusive. Here, we review the advances made by various studies in targeting multiple steps of signalling leading to IRF5 activation with their therapeutic potential, and the possible complications of such strategies are discussed.

**Abbreviations**
DBD: DNA‐binding domain
IAD: IRF‐associated domain
IBD: inflammatory bowel disease
IFN: interferon
iPSdMs: induced pluripotent stem cells
IRF5: interferon regulatory factor 5
ISREs: interferon‐stimulated response elements
LNA: locked nucleic acid
MHC: major histocompatibility complex
MI: myocardial infarction
MS: multiple sclerosis
ODNs: oligonucleotides
RA: rheumatoid arthritis
siRNA: small interfering RNA
SLE: systemic lupus erythematosus
TLR: toll‐like receptor

## Introduction

The interferon regulatory factor (IRF) family of transcription factors were first characterized for mediating antiviral responses and type I interferon (IFN) production. They have since been demonstrated to play diverse functions in apoptosis, the cell cycle, oncogenesis and gene regulation in response to pathogen‐derived signals 1. The mammalian IRF family comprise nine members (IRFs 1–9) with conserved multidomains 2. The N‐terminal DNA‐binding domain (DBD) recognizes a core DNA sequence within interferon‐stimulated response elements 3 and the C‐terminal IRF‐associated domain (IAD) mediates protein–protein interactions between IRFs and other proteins to form transcriptional complexes 4.

Among the IRF family members, interferon regulatory factor 5 (IRF5) plays a central role in inflammation. IRF5 mediates induction of proinflammatory cytokines such as interleukin‐6 (IL‐6), IL‐12, IL‐23 and tumour‐necrosis factor‐alpha (TNF‐α) 5, 6, and its recruitment to promoters of inflammatory genes is assisted by the NF‐kB p65 subunit RelA 7. IRF5 is a key factor in defining the inflammatory macrophage phenotype. IRF5 is highly expressed in not only monocytes and macrophages but also in B cells and dendritic cells. Its expression in macrophages can be upregulated in response to the inflammatory environment and, in particular, to the stimulation with GM‐CSF and IFN‐gamma 6, 8.

In humans, IRF5 exists as multiple distinct isoforms (V1–V11) that are generated by alternatively spliced transcripts. Each isoform utilizes one of three promoters, giving rise to transcripts containing exon 1A, 1B or 1C, and displays differences in composition of other exons. These isoforms show cell‐type specific expression, subcellular localization and function 9. Multiple GWAS studies have identified polymorphisms in the *Irf5* locus, leading to expression of alternatively spliced isoforms of *Irf5* that are associated with risk of autoimmune diseases such as systemic lupus erythematosus (SLE) in humans 10, 11. For example, IRF5 isoforms generated from exon 1B (v2, v9, v10) are strongly linked to overexpression of IRF5 and to susceptibility to SLE, whereas elevated expression of IRF5 in the absence of exon 1B does not confer risk 10. Several IRF5 isoforms including isoform v2 contain splicing variations in and around exon 6, which encodes for a proline‐, glutamic acid‐, serine‐ and threonine‐rich (PEST) domain thought to be important for protein stability in the IRF family of proteins 12.

## IRF5 as an attractive therapeutic target

There is overwhelming evidence that IRF5 plays a key role in numerous conditions based on the phenotype of IRF5 knockout mice in disease models. Mice lacking *Irf5* are resistant to lethal endotoxin‐induced shock with reduced expression of proinflammatory cytokines 5, 13. *Irf5*‐deficient mice are also protected from arthritis and lupus in murine models of inflammatory arthritis and pristane‐induced lupus respectively 14, 15, 16. For example, *Irf5* ^*−/−*^ mice exhibit reduced knee swelling when challenged with methylated BSA in the acute antigen‐induced arthritis murine model 16. *Irf5* ^*−/−*^ mice demonstrate impaired expression of IL‐12b and enhanced expression of IL‐10 in their affected joints 8. Moreover, proinflammatory monocyte‐derived macrophages with IRF5 expression are specifically detected in the affected knees. Somewhat contradictory to these results was a report describing no differences between WT and *Irf5* ^*−/−*^ mice in a model of collagen‐induced arthritis (CIA) 17. The mice on C57BL/6 background express the b haplotype of the major histocompatibility complex (MHC) class II and need MHC class II A(q) to develop CIA dependent on autoreactive T cells 18, 19. When C57BL/6 *Irf5* ^*−/−*^ mice were crossed with the strain carrying MHC class II Aq, a significant reduction in the number of mice developing the pathology was observed (H. Eames, unpublished data), suggesting that the conclusion of no role for IRF5 in the CIA induced pathologies needs to be revisited.

Increased IRF5 levels are associated with better prognosis of pulmonary disease 20. In murine asthma models with house dust mite (HDM) exposure, *Irf5* ^*−/−*^ demonstrate impaired lung function and extracellular matrix deposition, but mice overexpressing IRF5 were protected from allergic inflammation 20. Recent studies have also highlighted important contributions of IRF5 to neuropathic pain 21, vascular diseases 22, 23, 24, obesity 25 and hepatic and skin fibrosis 26, 27. For example, *Irf5* ^*−/−*^ mice on a high fat display beneficial expansion of subcutaneous adipose tissue and retain their insulin sensitivity 25. Other reported roles for IRF5 include cell cycle arrest and apoptosis 28, microbial infection 29, 30, and glycolysis 31. Several studies have also found that autoantibodies against IRF5 were able to cross react with homologous peptides from *Mycobacterium avium* subsp. *Paratuberculosis* and Epstein–Barr virus 32, 33, 34. Furthermore, antibodies against these peptides were significantly higher in the cerebrospinal fluid and serum of multiple sclerosis (MS) patients due to molecular mimicry 32, 33.

From these studies and given the fact that in humans *IRF5* gene polymorphisms related to higher *IRF5* expression 10, 35, 36, 37, 38, 39 have been associated with susceptibility to inflammatory and autoimmune diseases including rheumatoid arthritis (RA), inflammatory bowel disease, SLE, MS, and Sjörgens syndrome, IRF5 has emerged as an attractive target for therapeutic intervention.

A benefit of targeting IRF5, is that it acts in a cell‐type and activity‐specific manner. Both IRF5 and NF‐kB transcription factors are essential for the induction of proinflammatory genes 7. Due to the broader functional activities of NF‐kB and its ubiquitous nature, there are concerns of the detrimental effects that might result from blockage of NF‐kB activity. In comparison, targeting IRF5 may well be more beneficial and offer less adverse effects to general cell function.

Despite the strong rationale for targeting IRF5, inhibitors that interfere with the IRF5‐specific pathway remain elusive. This review provides an overview of some of the approaches currently used to target IRF5 and their potential as a therapeutic agent.

## Strategies in targeting IRF5

Given the complexity of IRF5 signalling, finding an effective and feasible way of targeting IRF5 function presents a challenge. Strategies for modulation of IRF5 activity and expression which will be discussed in further detail and include (a) Modulating IRF5 expression, (b) Interfering with the post‐translational modifications that modulate IRF5 function including phosphorylation and ubiquitination, and (c) Interfering with IRF5 association with protein partners, disrupting dimer formation or DNA binding. This review will describe the different approaches, the mechanism by which they affect IRF5 levels, considerations for their uses in therapeutic settings, and discuss their advantages and limitations (Table 1).

**Table 1:** Summary of strategies to study and modulate interferon regulatory factor 5 (IRF5) function

Mode of action	Class of drug	Advantages	Disadvantages
Inhibition of IRF5 gene expression	Small interfering RNA	Selectivity	Off‐target effects Delivery
Inhibition of IRF5 gene expression	CRISPR/Cas9	Easy design High efficiency	Off‐target effects
IRF5 overexpression	Adenoviral vector	High transduction efficiency High levels of transgene expression	Transient transfer and expression
Disruption of IRF5–protein interactions	Peptide inhibitors	Selectivity	Low proteolytic stability Low conformational stability
Disruption of IRF5–DNA interactions	Decoy oligonucleotides	Selectivity	Delivery
Inhibition of IRF5 kinases	Kinase inhibitors	Targetable by small molecule inhibitors	May interfere with other signalling pathways

### Modulating IRF5 expression

**Figure 1:**
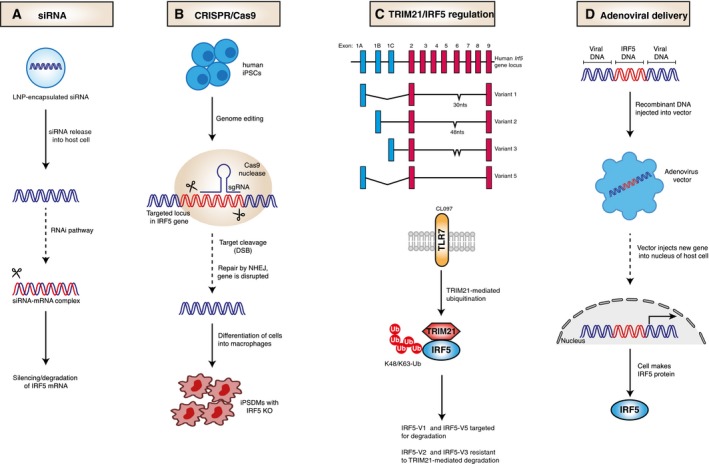
Strategies to modulate interferon regulatory factor 5 (IRF5) expression. (A) Small interfering RNA (siRNA)‐mediated therapy using lipidoid nanoparticles (LNP) delivery to modulate the expression of IRF5 through RNA interference (RNAi). (B) IRF5 knockouts in human‐induced pluripotent stem cell‐derived macrophages using the CRISPR/Cas9 system. (C) Top – splice variants of IRF5 including isoforms v1/v2/v3/v5. Each isoform includes exon 1A, 1B or 1C. Some isoforms have 30/48 nucleotide deletions in exon 6. Bottom – upon toll‐like receptor 7 (TLR7) stimulation TRIpartite motif 21 (TRIM21) regulates IRF5 stability and activity in an isoform‐specific manner. (D) IRF5 gene delivery with an adenoviral vector to increase levels of IRF5.

Several genetic manipulation technologies exist with the potential to control IRF5 expression levels and help reduce the severity of IRF5‐affected conditions (Fig. 1). Strategies for reducing IRF5 expression include small interfering RNA (siRNA) and locked nucleic acid (LNA) oligonucleotides (ODNs) for genetic knockdowns, or clustered regularly interspaced short palindromic repeats‐associated Cas9 nuclease (CRISPR‐Cas9) technology for genetic knockouts. These strategies are particularly useful for mechanistic and target validation experiments that will provide a platform for the development of specific therapeutic agents targeted towards IRF5, but could also be explored as therapeutic strategies themselves.

One approach to target IRF5 is to inhibit the expression of IRF5 using siRNA that are specific for sequences in the *irf5* mRNA (Fig. 1A). An *in vivo* study in an animal model of myocardial infarction (MI), demonstrated the use of siRNA to suppress IRF5 expression in cardiac macrophages 40. Intravenous administration of siRNA packaged lipidoid nanoparticles (LNP) in mice, attenuated M1 macrophage polarization with a decrease in M1 macrophage‐associated genes without increasing M2 genes. Furthermore, siRNA treatment ameliorated inflammation following MI, and improved infarct healing. Given that the delivery of siRNA‐encapsulated LNPs has proven safe and effective in humans, RNAi drugs could be a therapeutic prospect 41, 42, 43. Despite this, a number of hurdles must be overcome before RNAi drugs can be therapeutically used including off‐target effects, toxicity and delivery. To date, RNAi‐based therapeutics to modulate the expression of target proteins are currently at the clinical trial stages 44.

Related to siRNA‐mediated interference with target mRNA expression is the use of LNA‐based antisense ODNs. Although LNA to IRF5 is yet to be developed, of specific interest to this review is an attempt to target another transcription factor, HIF‐1α, in patients with refractory solid tumours which resulted in preliminary proof‐of‐concept results 45. The use of LNA antisense ODNs in human clinical studies have demonstrated proof‐of‐pharmacology for a number of targets 45, 46, 47, but have shown also some side effects 46.

A recent study utilized CRISPR/Cas9 to study the role of IRF5 in *Chlamydia* infection by generating IRF5 knockout mutations in macrophages derived from human‐induced pluripotent stem cells (iPSdMs; Fig. 1B) 48. *IRF5* iPSdM knockouts were more susceptible to *Chlamydia* infection, highlighting a role for IRF5 in limiting *Chlamydia* infection. Although the CRISPR‐Cas9 approach was used for mechanistic validation, the present study opens the possibility of achieving therapeutic genome editing of IRF5 in human macrophages. While still in its infancy, therapeutics using CRISPR/Cas9 are being developed. For example, genome editing by CRISPR to delete the *PD‐1* gene in T cells from lung cancer patients is reportedly underway 49.

The E3‐ubiquitin ligase TRIM21 has been demonstrated to target IRF5 for degradation and may potentially present another strategy based on modulation of IRF5 levels 50, 51. Following toll‐like receptor 7 (TLR7) activation, TRIM21 mediates degradation of IRF5 in an isoform specific manner, that is, it targets isoforms v1/v5 but isoforms v2/v3 are resistant (Fig. 1C). Increasing E3 activity of TRIM21 in macrophages may help to reducing the level of IRF5 in some but not all patients, as it will be ineffective against the isoform v2 linked to overexpression of IRF5 and to susceptibility to SLE 52.

Interferon regulatory factor 5 overexpression in the airway lumen was shown to enhance immune responses in the lung following allergen exposure 20. In this study, an adenoviral vector expressing IRF5 (AdIRF5) was used to increase IRF5 expression in the lungs (Fig. 1D). In response to HDM, IRF5 overexpression ameliorated airway hyper‐responsiveness, reduced mucus production and reduced goblet cell hyperplasia. Moreover, overexpression of IRF5 resulted in diminished production of type 2 cytokines and decreased eosinophilia. Thus, localized adenoviral delivery of IRF5 (e.g., via intranasal administration 20) to enhance IRF5 expression in macrophages and stimulate their immune potential could be a promising therapeutic strategy in eosinophilic asthma and would circumvent a concern related to the systemic administration of adenoviruses which results in hepatic tropism independent of the primary receptors 53.

Adenovirus‐based vectors are a widely used therapeutic platform for gene delivery, especially in the field of cancer gene therapy, where they demonstrate a good safety record and a great promise in preclinical studies 54. IRF5 overexpression could represent a promising approach to enhance cancer therapies through the reprogramming of tumour‐infiltrating macrophages, which are primarily M2‐polarized.

### Inhibiting IRF5 post‐translational modifications

**Figure 2:**
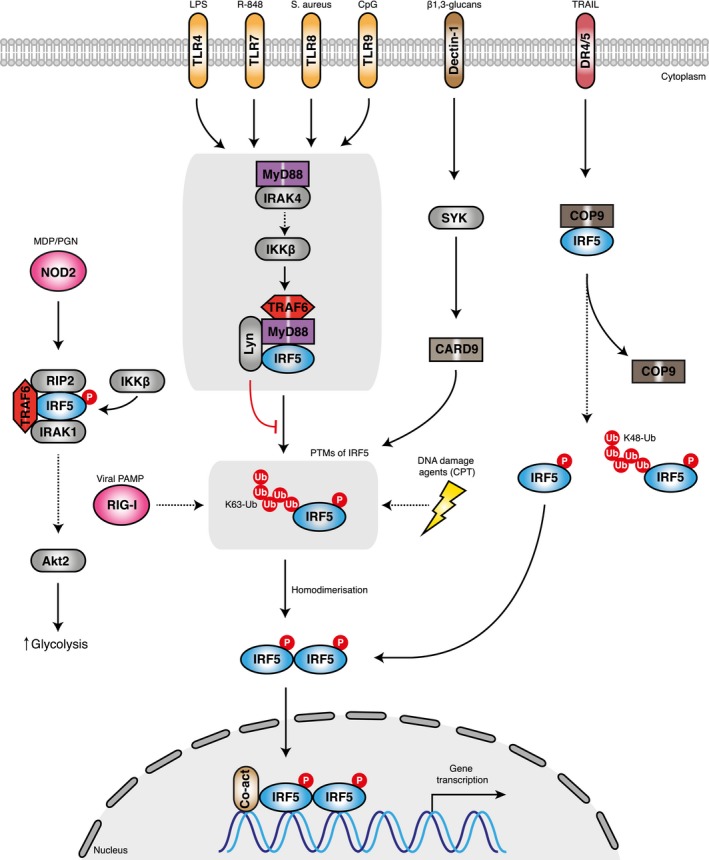
A schematic representation of the proposed mechanisms of IRF5 activation. The stimulation of toll‐like receptors (TLRs) on cell membranes by pathogen‐derived products or RIG‐I‐like receptors (RLRs) in the cytosol by viral PAMPs trigger signalling cascades. IRAK4 regulates the TLR/MyD88 response via TAK1 and IKKβ. Interferon regulatory factor 5 (IRF5) is activated from its latent state by post‐translational modifications that include ubiquitination by the ubiquitin ligase TRAF6 and phosphorylation by IKKβ. These modifications trigger IRF5 homodimerization, translocation to the nucleus and binding to gene promoters along with coactivator proteins (p300). Alternatively, DNA damage agents (CPT) can induce IRF5 phosphorylation and transcriptional activation. Another model proposes that the Dectin‐1‐induced IFN‐B production is mediated by a Syk‐Card9‐IRF5‐dependent pathway. In another model, IRF5 interacts with the COP9 signalosome (CSN). Upon stimulation with agonists such as TRAIL, an unknown kinase phosphorylates IRF5 which leads to its dissociation from CSN. Loss of the CSN–IRF5 interaction leads to K48‐linked ubiquitination of IRF5 and degradation by the ubiquitin–proteasome pathway. Some activated IRF5 migrates to the nucleus and activates target genes. An alternative model suggests that upon NOD2 stimulation IRF5 associates with RIP2, IRAK1, TRAF6 and IKKb to increase glycolysis through Akt2 activation.

Other than strategies to module IRF5 expression, for example, blocking the enzymatic action of regulators of IRF5 signalling including kinases and E3 ubiquitin ligases, could provide attractive targets for therapeutic intervention. The regulation of IRF5 has been reviewed in detail recently 55, and involves various signalling cascades that converge on IRF5 activation by ubiquitination, and phosphorylation (Fig. 2).

Probably most explored is the activation of IRF5 via the TLR‐MyD88 pathway 56. In this pathway, TLR activation by TLR ligands (LPS, R848, and CpG) induces a signalling cascade whereby IRF5 binds to TRAF6 and the adaptor protein MyD88 5, 57. Following TLR7/8 stimulation, IRAK4 kinase acts through TAK1 and IKKβ to activate IRF5 in human monocytes 58. A recent study found that Lyn kinase binds to and inhibits the activity of IRF5 in the TLR‐MyD88 pathway by blocking the post‐translational modification step in a kinase‐independent manner 59. Other modes of IRF5 activation include viral infection by Newcastle disease virus (NDV), vesicular stomatis virus (VSV) and herpes simplex virus type 1 (HSV‐1) 13, 60, 61, 62, as well as DNA‐damaging agents (CPT) 63.

Another study demonstrated a role for IRF5 in induction of IFN‐β following fungal infection by the pathogen *Candida albicans*, whereby the C‐type lectin receptor Dectin‐1 activation of IRF5 was dependent on the tyrosine kinase Syk and the adaptor protein Card9 30. An alternative model suggests that the COP9 signalosome (CSN) interacts with and stabilizes IRF5 64. Stimulation of the death receptor by TRAIL ligand leads to phosphorylation of the complex by an unknown kinase and IRF5 degradation through a ubiquitin–proteasome pathway. Some of the phosphorylated IRF5 migrates to the nucleus and transactivates target genes. IRF5 has been shown to associate with RIP2, IRAK1 and TRAF6 in human monocyte‐derived macrophages (hMDMs) 31. Each of these molecules along with IKKβ is required for NOD2‐induced IRF5 serine phosphorylation, which increases glycolysis through Akt2 activation 31.

During IRF5 signalling, IRF5 is subjected to TRAF6‐mediated K63‐linked ubiquitination 57. However, to date, there is some controversy in the literature on the functional consequence of this ubiquitination. Mutagenesis studies suggest ubiquitination of IRF5 is essential for nuclear translocation and target gene regulation 57. However, several lines of evidence using double KK/RR mutants, and the A20‐K63 ubiquitination enzyme have indicated that ubiquitination is not essential for IRF5 transcriptional activity 65. Instead, the carboxyl terminal phosphorylation of IRF5 is thought to be the critical modification that determines IRF5 transcriptional activity and thus a better therapeutic strategy is to target the kinase responsible for IRF5 phosphorylation.

**Figure 3:**
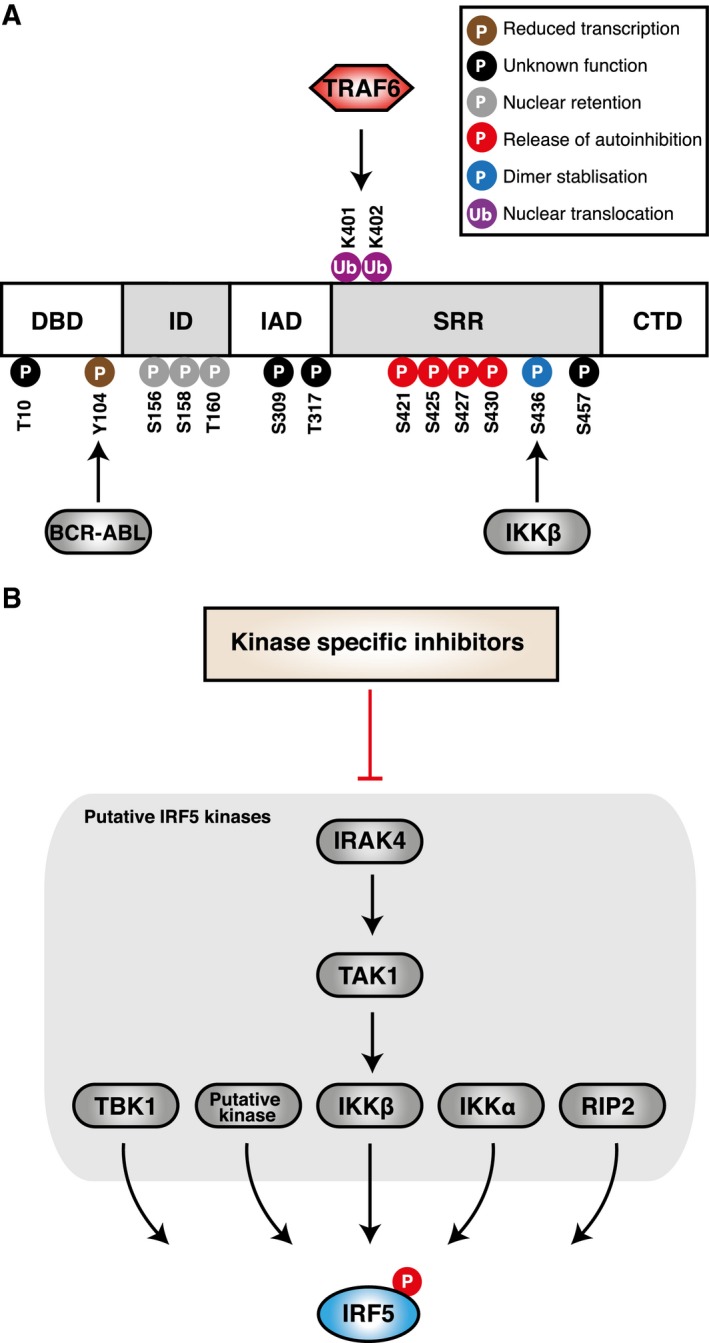
Modulation of interferon regulatory factor 5 (IRF5) via post‐translational modification. (A) Schematics of putative IRF5 phosphorylation and ubiquitination sites (human isoform v3/v4) and their positions with respect to functional domains. Suggested targets and functions of mapped sites are also shown (B) Inhibition of IRF5 phosphorylation through the use of kinase inhibitors. Kinases reported to phosphorylate IRF5 include IRAK4, TAK1, RIP2, IKKα, IKKβ, IKKε and TBK1.

Based on the crystal structure of the transactivation domain of pseudophosphorylated human IRF‐5, phosphorylation is thought to induce a conformational change in a C‐terminal autoinhibitory region, to enable dimerization 66. Phosphorylation sites towards the C‐terminal serine‐rich region of IRF5 include S425, S427, S430 and S436 (human isoform v3/v4); phosphorylation of S436 contributes to the stabilization of the activated dimer, whereas phosphorylation of S425, S427 and S430 are essential for release of the C‐terminal autoinhibitory conformation (Fig. 3A) 60, 66. Mass spectrometry analysis confirmed phosphorylation of S425 and S436 and revealed their contribution to IRF5 activity 65. Phosphomimetic S425, and S436D displayed an increase in IL‐12p40 promoter‐controlled luciferase activity. In contrast, the loss of function mutation with S436A reduced RIP2‐induced activation of the luciferase reporter, and the double mutant S427A, S436A also failed to induce promoter activity in cells stimulated with peptidoglycan (PGN) or muramyl dipeptide (MDP). Together, these studies indicate that phosphorylation of carboxyl serine residues are essential for IRF5 function, and provide a rationale for targeting the kinases involved.

### Kinase inhibitors

Targeting kinases in a signalling pathway with small molecule inhibitors is typically the most straightforward approach to block a protein. Kinases have been established as an important drug target. Despite a high degree of similarity in their catalytic core with the ATP‐binding pocket, kinases are amendable to blockade by small molecule agents with high selectivity. Moreover, high‐throughput kinase inhibitor libraries can identify potential candidate kinases. Several kinases including RIP2 from the receptor‐interacting protein (RIP) kinase family, and TAK1, IKKα, IKKβ, IKKε and TBK1 from the IKK family, are thought to phosphorylate IRF5 (Fig. 3B) 56, 67, 68. Phosphorylation of IRF5 by TBK1 or IKKε fail to induce nuclear translocation of IRF5 69. Furthermore, macrophages from TBK1‐deficient mice produce normal levels of proinflammatory cytokines in response to LPS 70. Thus, these kinases can be excluded as suitable IRF5 candidate kinases. Stimulation of the NOD2 intracellular receptor with MDP or PGN, can induce IFNB expression via IRF5 and RIP2 71. Using overexpression systems, a later study identified S436 on IRF5 as a RIP2 target residue 65. More recently, endogenous IRF5 serine phosphorylation in response to MDP was shown to be dependent on RIP2 and IKKβ, where IRF5 associates with RIP2, IRAK1 and TRAF6 31. IRF5 and IKKβ cross‐regulate each other's phosphorylation, but the kinase in the IRF5 complex responsible for IKKβ activation is unknown in this model 31. Since IKKB phosphorylation of IRF5 is known, it could be an interesting pharmacological target.

Earlier studies based on the use of IKKβ loss of function mutations, indicate that IKKβ activity is required for the activation of IRF5. Two groups have shown that phosphorylation by IKKβ on S436 induces IRF5 nuclear translocation and an IFN response 72, 73. Numerous inhibitors which are specific to IKKβ, and do not inhibit other IKK‐related kinases are available, but as IKKβ is involved in the activation of several targets its inhibition is unlikely to result in the specific inhibition of IRF5 function. Besides, the well documented role of IKKβ in phosphorylating IkB in the NF‐kB pathway, other reported substrates include forkhead transcription factor (FOXO3a), 14‐3‐3b, insulin receptor substrate 1 (ISR1) and docking protein 1 (DOK1) 74, 75, 76, 77. IKKα is another kinase from the IKK family reported to phosphorylate IRF5. Unlike IKKβ, IKKα‐mediated phosphorylation of IRF5 negatively regulates IRF5 in the MyD88‐IRF5 pathway 68. IKKα could represent a therapeutic target in conditions with low IRF5 activity. However, no potent commercially available IKKα‐specific inhibitors have been reported to date. Another reported kinase involved in IRF5 activation is TGF‐B‐activated kinase 1 (TAK1). Upstream of IKKβ, TAK1 can induce IFN‐B and IL‐12 in response to *Staphylococcus aureus* RNA 78. Several inhibitors for TAK1 reported to inhibit IRF5 include NG‐25, and 5Z‐7‐oxozeaenol, which both block nuclear translocation of IRF5 in monoyctes stimulated with TLR8 ligands 78. However, as TAK1 plays an essential role in several signalling pathways including the MAPK cascade and IKKs 79, inhibition of TAK1 might not offer the best approach for inhibiting IRF5 function.

In a recent report, IRAK4 was identified as a kinase that activates IRF5 via an IRAK4‐TAK1‐IKKβ axis in human monocytes 58. Chemical inhibition of IRAK4 with a potent and selective IRAK4 inhibitor blocked IRF5 nuclear translocation and IRF5 transcriptional activity at the promoters of inflammatory cytokines, such as IL‐1, Il‐6, TNF, in human monocytes. Despite blocking IKKβ activation, the IRAK4 inhibitor had no effect on NF‐kB nuclear translocation and transcriptional activity. In their proposed model, the kinase activity of IRAK4 kinase regulates the IRF5 pathway, whereas the scaffolding function of IRAK4 regulates the NF‐kB pathway. The requirement of IRAK4 kinase activity in regulating IRF5 supports the rationale for IRAK4 inhibitor use. Inhibition with IRAK4‐specific inhibitors could be sufficient to block IRF5 activity without having an impact on NF‐kB activation. This is a promising study and future studies will have to establish the efficiency of the inhibitor in disease mouse models. Mice harbouring the IRAK4 kinase‐dead mutant are protected from disease models of RA 80, emphasizing IRAK4 as an attractive therapeutic target in the context of IRF5.

### Interfering with IRF5‐interacting partners

Transcription factors are traditionally considered as ‘non‐druggable’ targets. Blocking their activity with small molecules presents a challenging task, given the large surface areas for binding proteins or DNA, the lack of hydrophobic pockets and the absence of enzymatic activity 81. Despite these obstacles, therapeutic modulation of transcription factor activity has been well documented by interfering with their ability to bind to DNA, partner proteins or their ability to dimerize. For example, the small molecule agent Nutlin can disrupt the p53–MDM2 interaction, thereby increasing p53 levels and p53 target gene expression 82. Another example is the MAML1‐derived stapled peptide which disrupts the NOTCH transcription complex and represses Notch transcriptional function 83.

One such method for blocking protein–protein interactions is the use of peptide inhibitors. These consist of short amino acid sequences that competitively inhibit the interaction between proteins. Several advantages that peptide inhibitors offer are their affordable synthesis, specificity, potency and activity 84. However, several drawbacks to consider are their low proteolytic stability and low conformational stability, which could decrease target binding 85. Despite these issues, ongoing efforts have been undertaken to develop effective peptide inhibitors to disrupt protein–protein interactions.

With regard to IRF5, peptide‐based inhibitors to decrease myocardial inflammation and fibrosis have been developed. In a murine model of systemic scleroderma, IRF5 bound to an apoAI mimic 4F, a peptide that inhibits myocardial inflammation 86. Hearts from 4F‐treated Tsk2/+ mice are less inflamed with a decrease in IRF5 expression, phosphoserine levels and nuclear localization. Whether these effects are mediated in part by the ability of 4F to directly bind to IRF5 or to other targets is unclear. It is also noted in the study that the changes observed may be due to differences in immune cell content. Nevertheless, the possible involvement of 4F binding to IRF5 warrants further exploration.

A more recent promising study developed a peptide inhibitor specifically designed from the peptide sequence of IRF5 23. The synthesized decoy peptide, termed IRF5D is derived from the IRF5 C‐terminal dimerization domain with the original sequence ELSWSADSIRLQISNPD replaced by the 17 amino acid‐long sequence ELDWDADDIRLQIDNPD. The aspartate substitution mimics activated IRF5, whereby IRF5D associates with IRF5 and prevents nuclear translocation of IRF5. In the study, Tsk/+ mice were used as a murine model of myocardial inflammation and fibrosis. IRF5D‐treated Tsk/+ mice produced reduced levels of ICAM‐1 and IRF5 expression in the heart, reduced leucocyte infiltration of the myocardium, improved endothelial vasodilation and IRF5 nuclear translocation was reduced in cultured Tsk/+ myocytes. Moreover, IRF5D treatment had no effect on endothelial cell (EC) proliferation and apoptosis. Thus, this study demonstrates that IRF5 is a druggable target and based on the published 3D structure of IRF5 66, it is possible to develop IRF5‐specific peptide inhibitors.

Alternatively, peptide inhibitors may target other interfaces such as the IRF5/RelA‐binding interaction. A previous study has demonstrated that IRF5 is recruited to the TNFα gene via interaction with NF‐kB subunit RelA 7, 87. The requirement for RelA for induction of proinflammatory genes with IRF5 supports the concept of developing inhibitors to block the IRF5–RelA interaction. This association has been mapped to the IAD of IRF5 and the dimerization domain (DD) of RelA 7.

Based on this information, along with the crystal structure of the IRF5 IAD 66, peptide inhibitors have been developed to sterically inhibit the IRF5/RelA interface but showed no significant inhibitory effects on IRF5‐dependent gene expression [88]. Further work to optimize peptide delivery and modifications to protect the peptide from degradation are worth pursuing to develop effective IRF5 inhibitors.

Other IRF5‐interacting proteins thought to be involved in IRF5 activation include CBP/p300 and histone deacetylases 67, 89, as well as signalling molecules KAP1, RIP2, TRAF6, MyD88, IRAK1 and IRAK4 5, 31, 57, 90 (Table 2). Thus, binding sites on IRF5 could be a potential peptide target and would require identifying the peptide sequences that are crucial for the IRF5–protein interactions. Another IRF5‐binding partner that has been shown to interact with and stabilize IRF5 is the CSN, which is thought to protect IRF5 from degradation by the ubiquitin–proteasome pathway 64. This interaction was mapped to the carboxyl and amino termini of IRF5. Disruption of the CSN–IRF5 interaction was shown to reduce IRF5 transactivation ability, whereas inhibition of IRF5 degradation by the proteasome enhances its transcriptional ability. Therefore, peptide inhibitors that target the CSN–IRF5 binding interaction could destabilize IRF5 and reduce IRF5 activity.

**Table 2:** Summary of the interferon regulatory factor 5 (IRF5) interactome

Negative gene regulation	Positive gene regulation	IRF5 activation
KAP1	RelA CBP/p300 Histone deacetylase	RIP2 TRAF6 MYD88 IRAK1 IRAK4 CSN LYN

The recent identity of Lyn from the Src family of tyrosine kinases as an IRF5‐binding partner that negatively regulates IRF5 in the TLR‐MyD88‐IRF5 pathway could be an interesting target to increase IRF5 activity in conditions such as asthma 20, 59. In a kinase‐independent manner, Lyn inhibits IRF5 by directly binding to IRF5 and preventing post‐translational modifications. Lyn deficiency in mice suffering from SLE leads to IRF5 hyperactivation, but reducing IRF5 levels ameliorates the disease development. Like IRF5, Lyn is highly expressed in immune cells (DCs, monocytes, macrophages and B cells), making it an attractive therapeutic target in inflammatory conditions. Since Lyn association with IRF5 is not activation or phosphorylation dependent, available Lyn allosteric activators such as MLR‐1023 91 are unlikely to affect IRF5 function. Instead, peptides could potentially bind and regulate IRF5 activation. The use of Lyn peptide inhibitors have been reported to bind to Lyn and block its association with the Bc receptor 92. *In vitro* the Lyn peptide inhibitor blocked Lyn‐dependent functions of IL‐5 92, and MD‐2 (Myeloid differentiation) tyrosine phosphorylation 93. Moreover, the peptide inhibitor blocked eosinophil differentiation, survival and airway influx in a murine model of asthma 92, 94. Therefore, peptide inhibitors that block the Lyn–IRF5 interaction could be used in the context of asthma to enhance IRF5 function. The Lyn–IRF5 binding interaction has been mapped to Lyns unique and kinase domains (LYN UD‐IRF5 DBD and Lyn KD‐IRF5 IAD) 59. Based on the amino acid sequence of these domains, peptides could be developed and tested for their ability to increase IRF5 activity.

An alternative approach is the use of ODNs containing the consensus binding site of a transcription factor. Use of decoy ODNs to bind to IRF5 offers another means to inhibit its activity, and has been recently been investigated 95. IRF5 and an ODN termed MS19 share a consensus AAAG repeat‐binding site. In a mouse model of septic peritonitis, MS19 treatment prolonged survival and reduced expression of iNOS, IL‐6 and TNF‐a, suggesting that it might be useful in treating inflammatory conditions. *In vitro* treatment of LPS‐stimulated RAW264.7 macrophages, also reduced expression of iNOS, IL‐6, TNF‐a and IRF5, with reduced nuclear IRF5 levels. MS19 is thought to bind to IRF5, prevent its nuclear translocation and subsequent induction of target genes. MS19 as a ODN to interfere with IRF5 function could be a potential therapeutic, that warrants further investigation. An efficient means to deliver MS19 to target cells, and its specificity towards IRF5 should be considered in future studies.

## Conclusions

There is overwhelming evidence that IRF5 plays a key role in physiology and disease, therefore dampening or enhancing IRF5 expression and activity provides new avenues for the development of therapeutic agents. Among the three strategies for modulation of IRF5 activity and expression discussed in this review, that is, (a) modulating IRF5 expression, (b) interfering with the post‐translational modifications of IRF5, and (c) interfering with IRF5 association with protein partners, the modulation of IRF5 levels using siRNA, CRISPR/Cas9, LNAs or perhaps nanobodies, single‐domain antibody fragments derived from camelid heavy‐chain antibodies that have been successfully utilized to target transcription factors 96, 97, 98, and adenoviruses, may take a long path in the development of new therapies themselves, but provide excellent opportunities for the identification of new points for therapeutic interference.

A more promising strategy at this stage is the use of specific inhibitors to the components of the IRF5‐signalling pathway, for example, kinase inhibitors. An ideal kinase inhibitor should block IRF5 function with limited effects on other signalling pathways. Thus, targeting IKKβ or TAK1 to block IRF5 activity are not ideal approaches, as these kinases target multiple pathways and may produce unwanted side effects. The selectivity of the IRAK4 inhibitor on the IRF5 pathway appears to carry a great potential. Future studies using high‐throughput libraries of kinase inhibitors may help identify novel kinases involved in IRF5 activation.

Another therapeutically promising approach is the use of peptides or small molecules to disrupt interactions with IRF5 cognate partners. The peptide inhibitors or small molecules that block the Lyn–IRF5 interaction could be used in the context of asthma to enhance IRF5 function. In fact, Lyn peptide inhibitors has already been shown to have a beneficial effect in a murine model of asthma 92, 94 and requires further assessment in human setting. Similarly, developing inhibitors or small molecules that could block the IRF5 interactions with RelA or the CSN, would be beneficial for suppressing the unwanted proinflammatory macrophage gene programme in inflammatory diseases.

Although there is still a long way to go, there is reason to believe that eventually some of the strategies discussed here will form the basis of effective therapies in IRF5‐associated conditions.
